# Congenital Affections of the Heart

**Published:** 1894-12

**Authors:** 


*Congenital Affections of the Heart.* By GEORGE CARPENTER, M.D. Pp. 103. London: JOHN BALE & SONS. 1894.

This little book gives a very good account of an obscure subject. It is clearly written, and the author shows a thorough acquaintance with his subject, due to a considerable experience in these affections. He does not attempt in this short monograph to compete with the larger treatises on the subject; but we think that he has been successful in writing a very useful and practical little book, just adapted to the wants of the student and practitioner. A short account is given of the developmental anatomy of the heart and great vessels; then the different forms of malformation are described, there is a section on symptoms, prognosis and treatment, and the clinical histories of some well-selected cases are added. The author very rightly points out that the murmurs present in these cases cannot be relied upon for an exact diagnosis of the condition present, and cautions his readers against concluding, as is so commonly done, that all cardiac bruits occurring under four years of age are of congenital origin. He gives some valid reasons for thinking that a certain percentage may be due to rheumatism. We think the book a good one; but why has it neither table of contents nor index?

